# Variation in the Distribution Characteristics of *Nemopilema nomurai* in Relation to Marine Environmental Conditions

**DOI:** 10.3390/biology15070570

**Published:** 2026-04-02

**Authors:** Sunyoung Oh, Kyoung Yeon Kim, Seok Hyun Youn, Kyounghoon Lee

**Affiliations:** 1 Oceanic Climate and Ecology Research Division, National Institute of Fisheries Science, Busan 46083, Republic of Korea; ohsunyoung1@naver.com (S.O.); weedy7411@korea.kr (K.Y.K.); younsh@korea.kr (S.H.Y.); 2 Division of Marine Production System Management, Pukyong National University, Busan 48513, Republic of Korea

**Keywords:** *Nemopilema nomurai*, marine environmental factors, acoustic survey, vertical distribution

## Abstract

In this study, we examined the interannual distribution patterns of the giant jellyfish *Nemopilema nomurai* and their relationship with marine environmental factors using field survey data collected from the East China Sea from 2020 to 2024. Acoustic surveys and jellyfish-specific trawl sampling were conducted to analyze their vertical distribution characteristics in relation to water temperature, salinity, seawater density, and chlorophyll-*a* concentrations. The results revealed that *N. nomurai* were mainly distributed in the mid-to-lower water layers at depths of 40–60 m, particularly in areas with strong stratification between the surface and bottom waters. Temperatures and salinity at these depths were relatively stable, and jellyfish were concentrated within high-density layers (1024.6–1025.0 kg·m^−3^). Correlation analysis revealed significant positive relationships between jellyfish occurrence frequency and salinity and seawater density, whereas no significant relationship was observed with water temperature. The chlorophyll-*a* concentrations varied between the years but did not directly correspond to the primary habitat depths of jellyfish, although synchronous variations in jellyfish abundance occurred in some years. These results indicate that the vertical distribution of *N. nomurai* is primarily controlled by physical oceanographic factors rather than by chlorophyll-*a* concentrations, reflecting an ecological adaptation for efficient energy use.

## Simple Summary

Large outbreaks of jellyfish populations can pose considerable challenges to fisheries, coastal activities, and marine ecosystems. To comprehensively elucidate why these outbreaks occur, we investigated the habitats of giant jellyfish *Nemopilema nomurai* in the ocean and the changes in their distribution with varying environmental conditions. Using data from field surveys conducted in the East China Sea from 2020 to 2024, we examined how the depth distribution of jellyfish is affected by seawater temperatures, salt content, water density, and food availability, as indicated by microscopic plants in the water. We found that jellyfish were most concentrated at middle depths rather than near the surface, particularly in areas where ocean water formed stable layers. Jellyfish tended to stay in water layers with a density similar to that of their own bodies, which helped them save energy while swimming. Although areas with high food availability did not directly match jellyfish depth, they appeared to indirectly support jellyfish populations by providing favorable feeding conditions. These findings enhance our understanding of how jellyfish use the oceanic environment and why they gather at specific depths. This knowledge can help scientists and managers better predict jellyfish outbreaks and reduce their future impacts on fisheries, coastal industries, and marine ecosystems.

## 1. Introduction

Jellyfish are widely distributed across global marine environments, with more than 2000 species documented to date. However, only a subset of these species has been taxonomically described, and new species continue to be identified and classified [1]. Jellyfish populations often exhibit substantial fluctuations in response to environmental conditions, and rapid increases in abundance-commonly referred to as blooms-have been reported in numerous regions [2,3].

Jellyfish blooms are closely associated with environmental variability driven by climate change, while anthropogenic pressures such as eutrophication and overfishing have also been recognized as major contributing factors [4,5]. Furthermore, climate-induced alterations in marine environmental conditions can influence the distribution, abundance, and population dynamics of jellyfish [6], raising growing ecological and socio-economic concerns, particularly in coastal and semi-enclosed seas. Consequently, identifying the environmental factors that regulate jellyfish distribution has become increasingly important.

Jellyfish that occur in high densities constitute important components of marine ecosystems, affecting trophic structure and biogeochemical cycling [7]. Their distribution and movement are strongly shaped by physical environmental gradients, including temperature, salinity, and light availability [8]. Although previous research has largely focused on broad-scale distribution patterns and bloom dynamics, comparatively limited attention has been directed toward the vertical (depth-related) distribution of jellyfish. In particular, the influence of depth-dependent environmental gradients on jellyfish occurrence and abundance remains insufficiently understood. Because vertical distribution plays a critical role in habitat use and aggregation behavior, elucidating how environmental conditions vary across depth layers is essential for understanding jellyfish habitat selection.

Therefore, this study investigates the relationship between marine environmental factors and the vertical distribution of *Nemopilema nomurai*, a species known to have substantial impacts on fisheries and marine ecosystems due to its large-scale outbreaks. In particular, we focus on identifying the depth range where jellyfish are most abundant. By clarifying these depth-specific habitat preferences, this study provides insight into how changing marine environmental conditions influence the vertical distribution of jellyfish.

## 2. Materials and Methods

### 2.1. Survey Area

**Figure 1:**
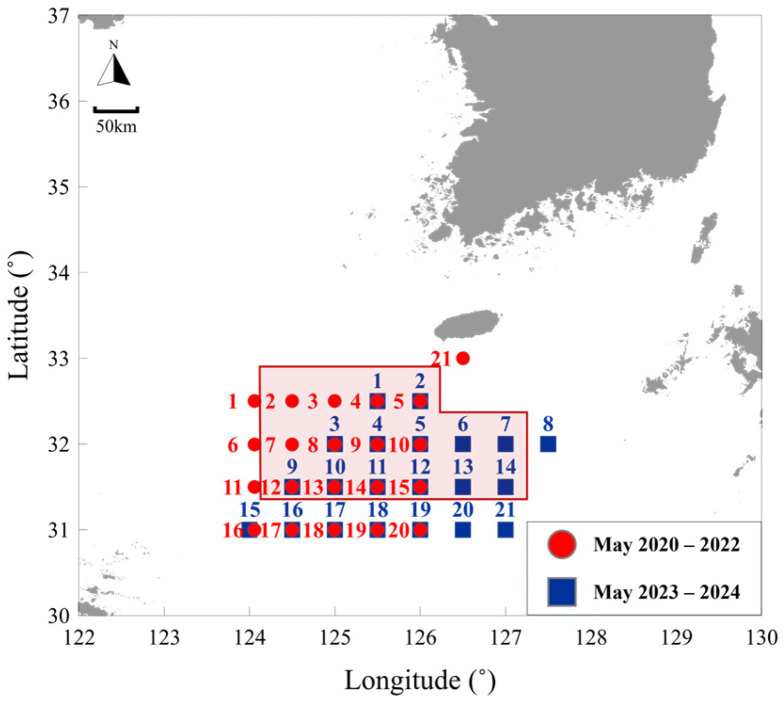
Survey stations for *N. nomurai* in the East China Sea from May 2020 to May 2024; 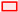: Stations overlapping with NIFS serial Oceanographic observation, 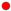: *N. nomurai* survey stations conducted from 2020 to 2022, 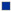: *N. nomurai* survey stations conducted from 2023 to 2024.

**Figure 2:**
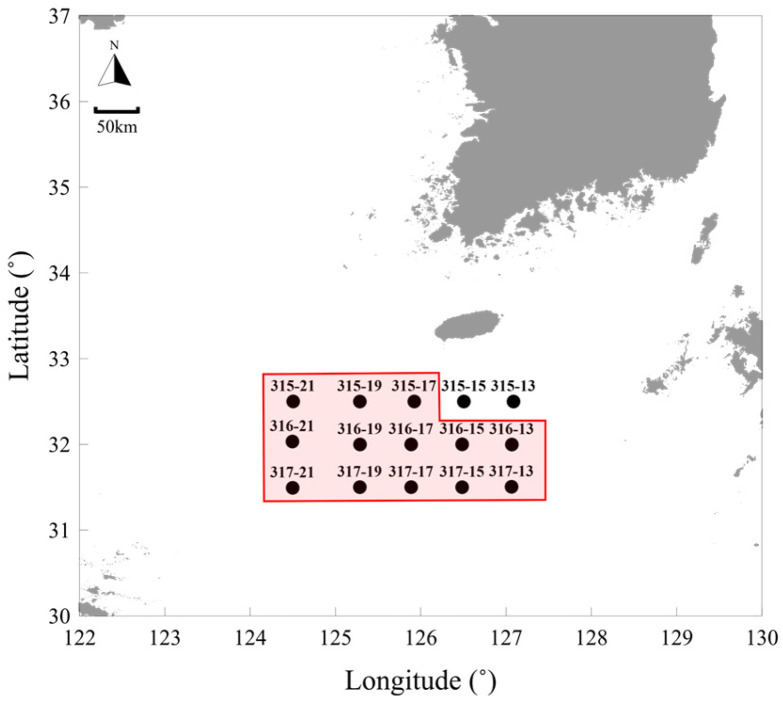
NIFS serial oceanographic survey stations conducted to collect marine environmental data in the East China Sea from May 2020 to 2024; 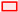: Stations overlapping with *N. nomurai* survey.

In this study, we collected data through annual surveys conducted from May 2020 to May 2024 in the East China Sea. For comparison with environmental data, we utilized serial oceanographic investigation data from the National Institute of Fisheries Science (NIFS). Serial oceanographic data were collected annually in February, May, August, and November. Herein, the May observation data were considered to correspond to those from the jellyfish surveys. To compare the jellyfish survey data with the serial oceanographic investigation data, those stations where the sampling points of the two datasets overlapped were analyzed separately (Figure 1 and Figure 2).

### 2.2. Acoustic Data Collection and Analysis

In 2020, acoustic surveys were conducted using the R/V Tamgu 8 (282 G/T). As an acoustic equipment was not permanently installed on this vessel, a split-beam scientific echosounder (EK-60; Simrad; Kongsberg Maritime AS, Kongsberg, Norway) operating at frequencies of 38 and 120 kHz was mounted on the side of the ship to collect acoustic data. For the remaining survey years, split-beam scientific echosounders (EK-80; Simrad; Kongsberg Maritime AS; Kongsberg; Norway) permanently installed on the hulls of R/V Tamgu 20 (885 G/T) and R/V Tamgu 21 (999 G/T), operated by NIFS, were used to collect acoustic data at frequencies of 18, 38, 70, 120, 200, and 333 kHz. All acoustic systems were calibrated using a standard tungsten carbide sphere prior to deployment. To ensure interannual comparability, acquisition settings and data processing criteria were kept consistent across surveys, and care was taken to minimize potential variability associated with differences in transducer mounting.

The acoustic data collected in the field were processed and analyzed in the laboratory using acoustic post-processing software (Echoview v8.0; Echoview Software Pty Ltd.; Hobart; Australia). To remove impulse noise collected during surveys and isolate jellyfish acoustic signals, we used data from the 38 kHz and 120 kHz frequencies to extract jellyfish echoes.

**Figure 3:**
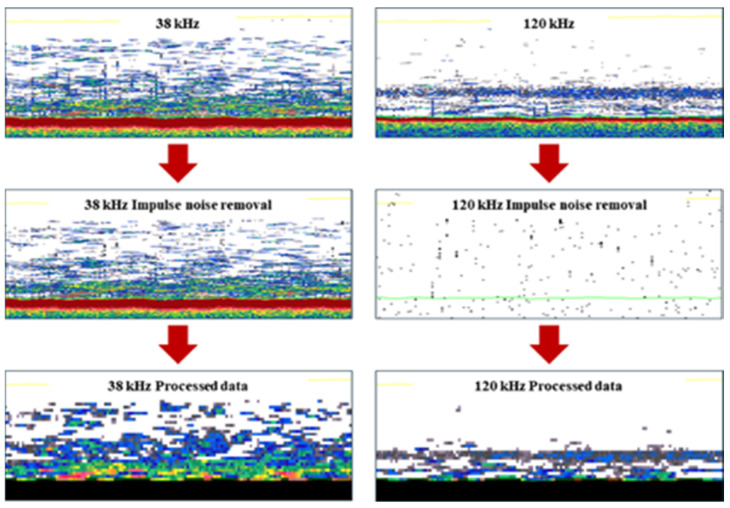
Flowchart of the process for removing impulse noise from data collected using a scientific echosounder; the process from raw data to noise removal.

When collecting acoustic data using scientific echosounders, various types of acoustic noise can cause the loss or distortion of valid echo signals. In this study, impulse noise was removed by applying a threshold value of −150 dB, following the method described by [9]. A flowchart illustrating the noise processing procedure applied to the acoustic data is shown in Figure 3.

To isolate jellyfish acoustic signals, it is necessary to identify the frequency characteristics and differences between the 38 kHz and 120 kHz frequencies. Here, the frequency difference refers to the difference in the mean volume backscattering strength between multiple frequencies, which was calculated by comparing the target strength (TS) values of the target organism at different frequencies and subtracting the lower TS value from the higher one.

When extracting the jellyfish acoustic signals using the frequency difference method, the previously reported [10] acoustic backscattering strength values were applied. In addition, the frequency difference ranges corresponding to the jellyfish size were determined based on jellyfish specimens collected through trawl surveys conducted in the field. The resulting frequency difference ranges applied in the acoustic surveys are listed in Table 1. All signals other than those identified as potential jellyfish echoes were classified as noise and removed, after which a single-target detection method was applied.

**Figure 4:**
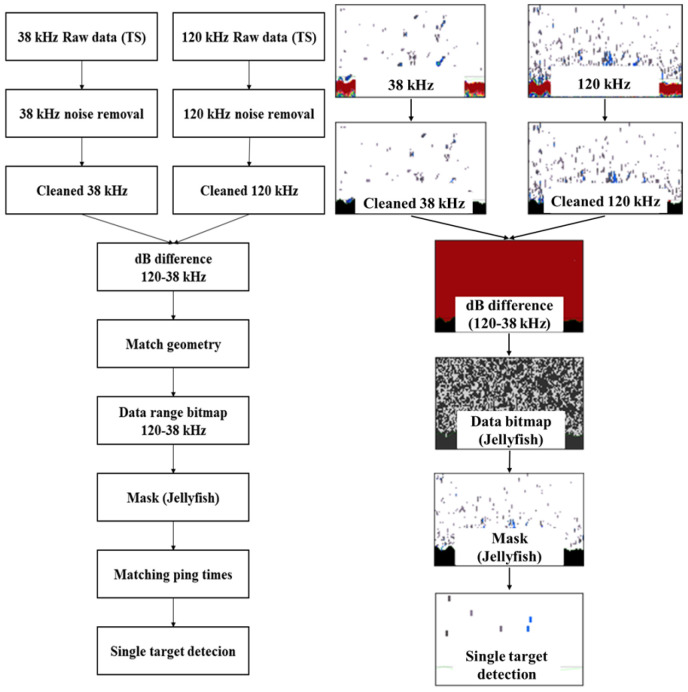
Flowchart for identifying *N. nomurai* using acoustic data at 38 kHz and 120 kHz frequencies; Process from raw data to single target detection.

The single−target detection method is an algorithm-based approach designed to detect and identify individual swimming targets independently. The parameter values used to extract the jellyfish acoustic signals are summarized in Table 2, and the overall procedure is illustrated in Figure 4. The single-target detection method is an algorithm-based approach designed to detect and identify individual swimming targets independently. The parameter values used to extract the jellyfish acoustic signals are summarized in Table 2, and the overall procedure is illustrated in Figure 4. The TS threshold (−65 dB) was determined based on previously reported size-dependent TS relationships for *N. nomurai* and was applied as a representative and conservative threshold for reliable jellyfish detection.

### 2.3. Collection of Marine Environmental Data

To compare environmental conditions, seawater temperature and salinity were measured at multiple depths using a CTD (Conductivity–Temperature–Depth) profiler (SBE 911plus; Sea-Bird Electronics; Bellevue, WA, USA), a widely used instrument for acquiring essential oceanographic variables [11]. Such hydrographic data provide fundamental information for understanding marine environments and are increasingly integrated into spatial and ecosystem-based analyses [12].

Seawater samples for chlorophyll-*a* measurements were collected at discrete depths (0, 10, 20, 30, 50, 75, and 100 m) by using a rosette sampler installed in the research vessel. Seawater samples collected from the field were filtered through Whatman GF/F filters, frozen, and transported to the laboratory. Chlorophyll-*a* was extracted using acetone and quantified using a fluorometer (10-AU Fluorometer; San Jose, CA, USA).

In this study, the total chlorophyll-*a* concentration results, including those for all size fractions, were compared with the acoustic survey results.

### 2.4. Seawater Density Analysis

To examine seawater density in the study area, calculations were performed using the TEOS-10 (Thermodynamic Equation of Seawater 2010) framework. TEOS-10 is an internationally standardized formulation developed to calculate the thermodynamic properties of seawater. It enables the accurate estimation of various physical properties of seawater, including density, heat capacity, and conductivity, based on variables such as temperature, salinity, and depth. The TEOS-10 replaced the previously used Practical Salinity Scale (PSS-78) and Equation of State 1980 (EOS-80), allowing for a more precise investigation of seawater properties in oceanographic research.

In the TEOS-10 framework, seawater density (ρ, kg·m^−3^) is calculated using absolute salinity (S_A_, g·kg^−1^), conservative sea temperature (θ, °C), and pressure (p, dbar). Thus, seawater density was calculated using Equation (1) as follows:

(1) $$\rho =\rho (S_{A}\theta ,P)$$

This formulation was implemented using the TEOS-10 algorithm in the Gibbs SeaWater (GSW) Oceanographic Toolbox for R.

## 3. Results

### 3.1. Relationship Between Marine Environmental Characteristics and the Vertical Distribution of N. nomurai from 2020 to 2024

**Figure 5:**
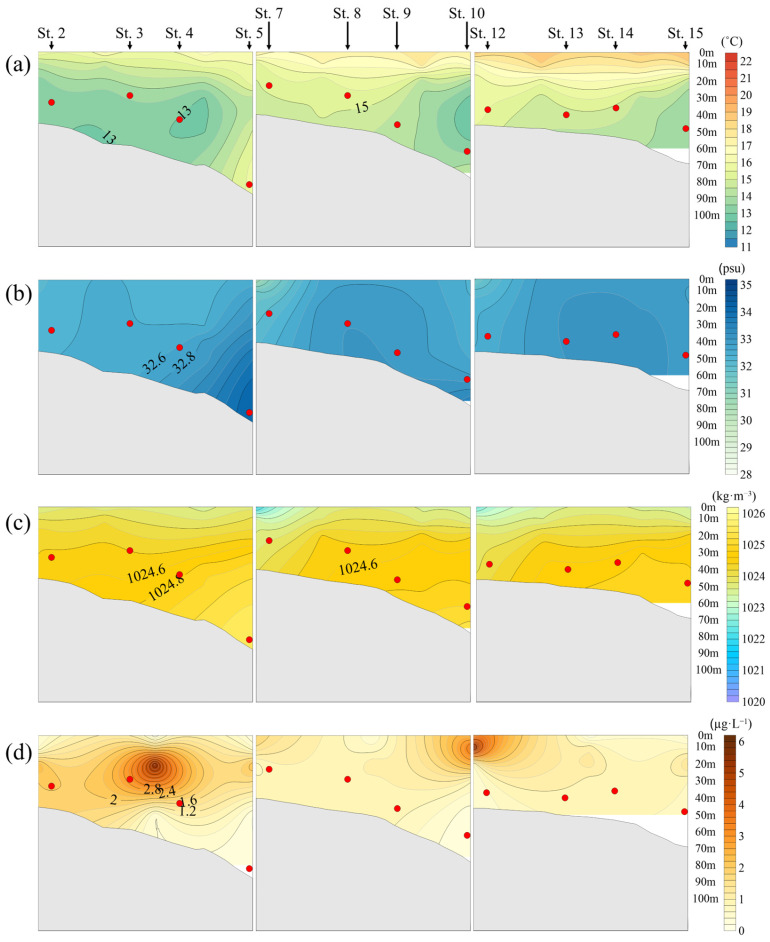
Relationship between *N. nomurai* distribution depth and vertical distribution according to the marine environment in the overlapping sections of jellyfish survey stations and NIFS serial Oceanographic observation in 2020: (**a**) Temperature (°C); (**b**) Salinity (psu); (**c**) Seawater density (kg·m^−3^); (**d**) Chlorophyll-*a* (μg·L^−1^); 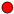: depth of *N. nomurai* distribution.

In May 2020, jellyfish were mainly distributed at a depth of approximately 40 m, with an average distribution temperature of 14.2 °C. The vertical temperature profile showed a sharp change in the mid-water layer (Figure 5a).

Salinity showed relatively low values at the surface and increased with depth (Figure 5b). This vertical structure of temperature and salinity was reflected in the density distribution, with low-density water at the surface and high-density water in deeper layers. Jellyfish were mainly distributed within these relatively high-density layers (Figure 5c).

Chlorophyll-*a* concentrations showed high values at the surface between Station 5 and Stations 3–4, and decreased with increasing depth. In particular, relatively high concentrations were observed at depths of 10–20 m (Figure 5d).

**Figure 6:**
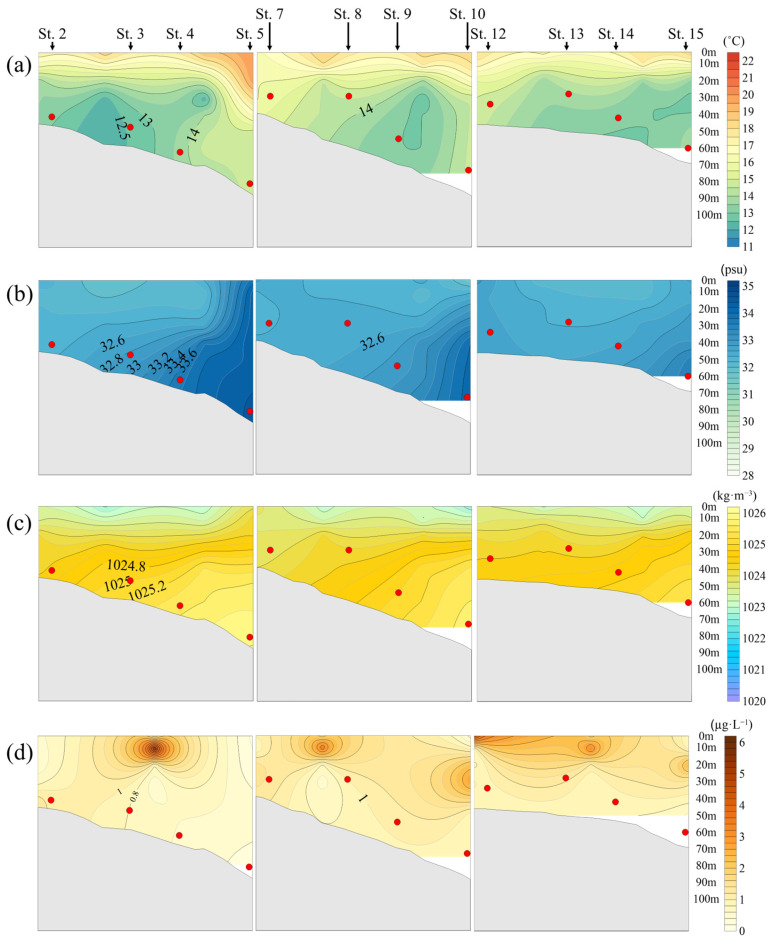
Relationship between *N. nomurai* distribution depth and vertical distribution according to the marine environment in the overlapping sections of jellyfish survey stations and NIFS serial Oceanographic observation in 2021: (**a**) Temperature (°C); (**b**) Salinity (psu); (**c**) Seawater density (kg·m^−3^); (**d**) Chlorophyll-*a* (μg·L^−1^); 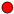: depth of *N. nomurai* distribution.

In 2021, jellyfish were mainly distributed in the mid-to-lower layers, within a temperature range of 12.3 °C to 16.0 °C, with an average distribution temperature of 14.1 °C (Figure 6a).

The salinity at the depths where jellyfish were mainly distributed was similar to that observed in 2020, ranging from 32.2 psu to 34.5 psu, with an average of 33.1 psu. High-salinity layers were formed in the eastern part of the study area due to the influence of warm currents (Figure 6b).

Jellyfish were mainly distributed within a density range of 1023.9 kg·m^−3^ to 1026.0 kg·m^−3^, with an average of 1024.9 kg·m^−3^, indicating their occurrence in relatively high-density layers. Low-density layers were formed at the surface due to low-salinity water masses, while high-density water masses were formed at depths of 40–80 m (Figure 6c).

Chlorophyll-*a* concentrations were high at 10 m at Stations 3–4 and Station 8, and elevated concentrations were observed between 20 and 30 m at Stations 10 and 15. Overall, the vertical distribution pattern was similar to that in 2020 (Figure 6d).

**Figure 7:**
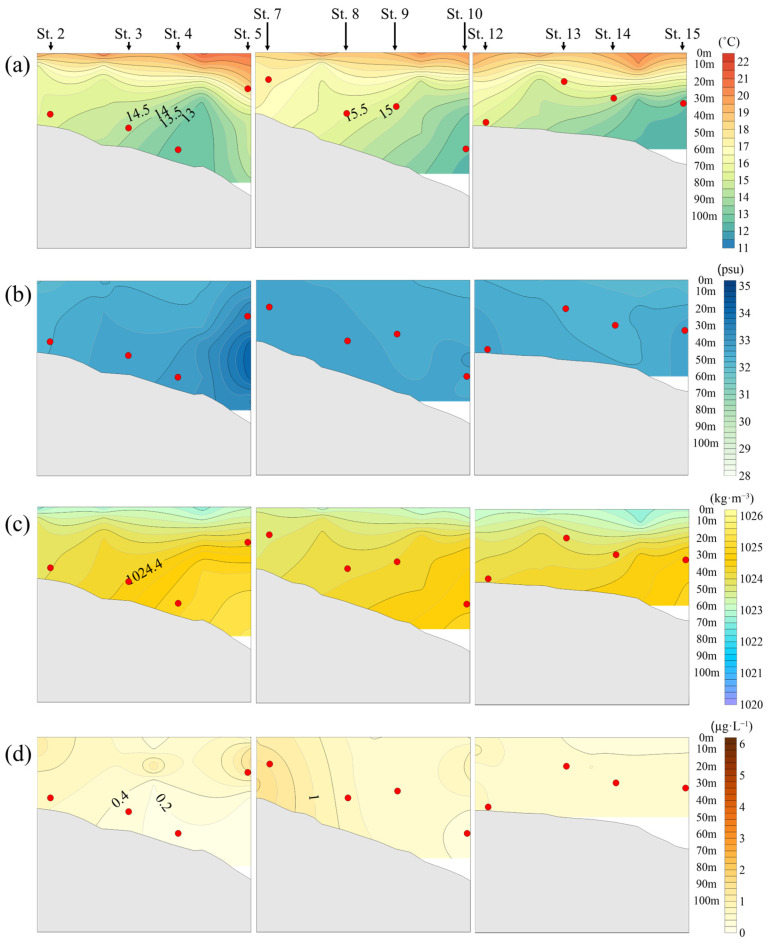
Relationship between *N. nomurai* distribution depth and vertical distribution according to the marine environment in the overlapping sections of jellyfish survey stations and NIFS serial Oceanographic observation in 2022: (**a**) Temperature (°C); (**b**) Salinity (psu); (**c**) Seawater density (kg·m^−3^); (**d**) Chlorophyll-*a* (μg·L^−1^); 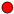: depth of *N. nomurai* distribution.

The temperature at the depths where jellyfish were mainly distributed ranged from 12.5 to 19.1 °C, with an average of 14.8 °C. This represented the highest average temperature during the study period, and in 2022, surface water temperatures generally showed an increasing trend. In the eastern part of the study area, where the influence of freshwater was relatively low, a low-temperature water mass of 12–13 °C was formed in the bottom layer (Figure 7a).

The salinity at the depths of jellyfish distribution had an average value of 32.7 psu. High-salinity water masses were formed due to the influence of saline water inflow from offshore areas (Figure 7b).

The density at the depths where jellyfish were mainly distributed had an average of 1024.3 kg·m^−3^. At stations adjacent to the Yangtze River, low-salinity water mixed with relatively high-temperature water, forming low-density water masses in the surface layer, while in the eastern part of the East China Sea, high-density water masses were formed in the bottom layer due to the combination of low temperature and high salinity (Figure 7c).

Chlorophyll-*a* concentrations decreased sharply in 2022, showing reductions of approximately 52% and 48% compared to 2020 and 2021, respectively. Relatively high concentrations were observed at Station 7, which is influenced by the discharge from the Yangtze River (Figure 7d).

**Figure 8:**
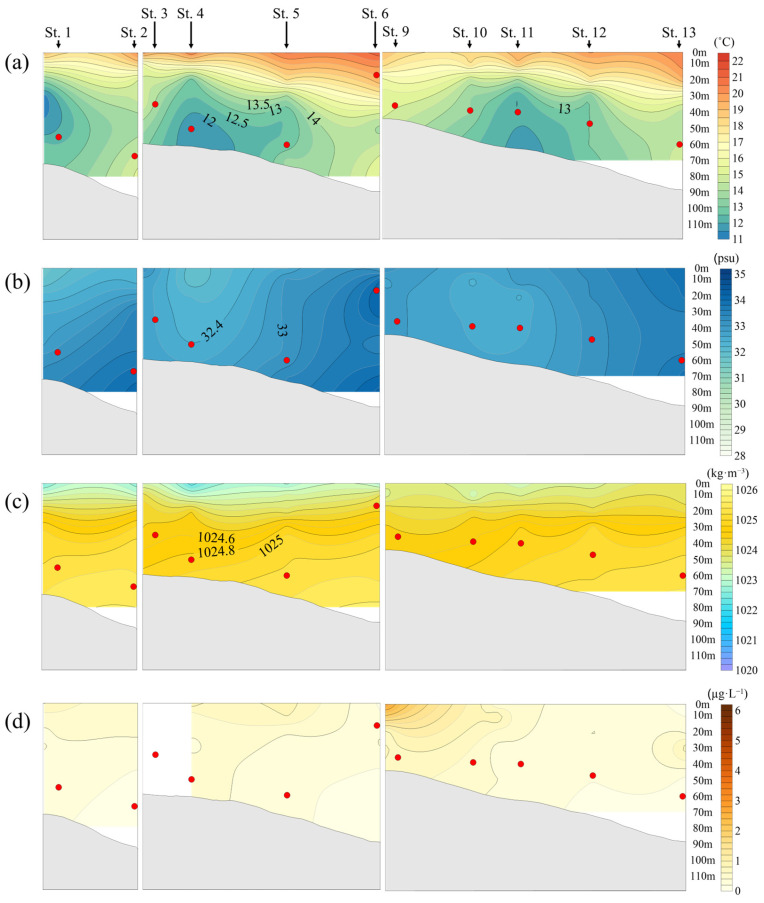
Relationship between *N. nomurai* distribution depth and vertical distribution according to the marine environment in the overlapping sections of jellyfish survey stations and NIFS serial Oceanographic observation in 2023: (**a**) Temperature (°C); (**b**) Salinity (psu); (**c**) Seawater density (kg·m^−3^); (**d**) Chlorophyll-*a* (μg·L^−1^); 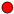: depth of *N. nomurai* distribution.

In 2023, jellyfish were mainly distributed at depths of approximately 40 m. The temperature range was the widest over the five-year period, and the maximum temperature was the highest, with an average of 13.9 °C. Considering only the depths where jellyfish were distributed, they tended to occur in relatively lower temperature ranges compared to the previous year (Figure 8a).

The salinity at the main jellyfish distribution depths ranged from 32.4 to 34.1 psu, with an average of 33.1 psu, showing a distribution pattern similar to that observed in 2022 (Figure 8b).

The density at the main jellyfish distribution depths had an average of 1024.9 kg·m^−3^. As depth increased, high-density water masses were formed in the bottom layer, while low-density water masses formed by the influence of freshwater discharge from the Yangtze River were distributed in the surface layer, showing a clear boundary with the surrounding water masses (Figure 8c).

Chlorophyll-*a* concentrations showed the lowest levels over the five-year period. Although relatively higher concentrations were observed at depths of 10–30 m, overall low concentrations were maintained throughout the water column (Figure 8d).

**Figure 9:**
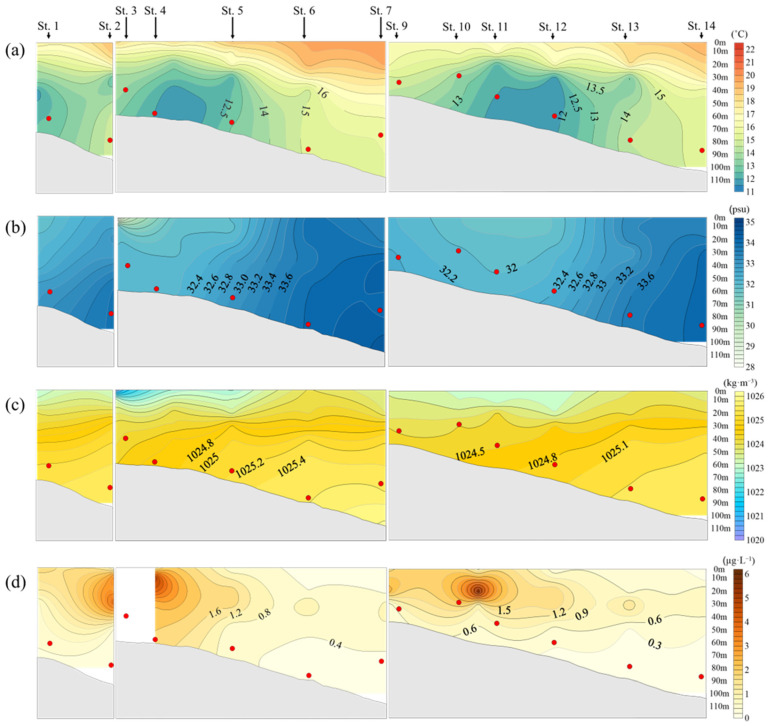
Relationship between *N. nomurai* distribution depth and vertical distribution according to the marine environment in the overlapping sections of jellyfish survey stations and NIFS serial Oceanographic observation in 2024: (**a**) Temperature (°C); (**b**) Salinity (psu); (**c**) Seawater density (kg·m^−3^); (**d**) Chlorophyll-*a* (μg·L^−1^); 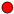: depth of *N. nomurai* distribution.

In 2024, jellyfish were mainly distributed at depths of approximately 60 m. The temperature at the main jellyfish distribution depths ranged from 11.7 to 15.5 °C, with an average of 13.7 °C (Figure 9a).

The salinity at the depths where jellyfish were distributed had an average of 33.0 psu, showing a similar range to that observed in 2023 (Figure 9b).

The density at the depths where jellyfish were mainly distributed ranged from 1024.1 to 1025.6 kg·m^−3^, with an average of 1025.0 kg·m^−3^. Low-density water was observed in the surface layer due to the influence of high temperature and low salinity, and a clear separation between the surface and bottom layers was formed (Figure 9c).

Chlorophyll-*a* concentrations showed the highest levels over the five-year period, increasing by approximately 20%, 29%, 149%, and 171% compared to 2020, 2021, 2022, and 2023, respectively. High chlorophyll-*a* concentrations were mainly observed in areas influenced by discharge from the Yangtze River (Figure 9d).

### 3.2. Comprehensive Analysis of Interannual Variations in Marine Environmental Conditions and N. nomurai Distribution

The relationship between interannual variations in marine environmental factors and the vertical distribution depth of jellyfish is summarized in Table 3. Over the 5-year experimental period, mean water temperatures ranged from 13.7 °C to 14.8 °C, whereas the temperatures at depths where jellyfish were predominantly distributed ranged from 11.6 °C to 19.7 °C. The highest temperatures are observed in 2022, whereas relatively low temperatures are observed in 2023 and 2024. A clear stratification between the surface and bottom layers was consistently observed. Jellyfish were mainly active in the mid-water layer (40–60 m) and were predominantly distributed in areas where mixing between the surface and bottom waters was weak.

The mean annual salinity ranged from 32.1 to 33.1 psu. The highest salinity value (34.5 psu) was recorded in 2021, which likely reflects the strong influence of high-salinity water masses intruding from offshore regions. In contrast, lower salinity values were observed in 2020 and 2024, when freshwater discharge from the Yangtze River had a stronger influence. Low-salinity water masses formed in the surface layer, resulting in a tendency for the jellyfish habitat range to be restricted to the mid- and lower water layers.

Seawater density ranged from 1023.7 kg·m^−3^ to 1026.0 kg·m^−3^, whereas densities at the primary jellyfish distribution depths ranged from 1024.6 kg·m^−3^ to 1025.0 kg·m^−3^, indicating that jellyfish were generally distributed within relatively high-density layers. In particular, in 2023 and 2024, high-density water masses formed in the bottom layer, whereas low-density water layers were present at the surface, creating a pronounced stratified structure. These density differences were closely associated with the variations in temperature and salinity and played a key role in determining the primary depth range of jellyfish habitats.

The chlorophyll-*a* concentrations were the highest in 2024 (5.98 µg·L^−1^), representing an increase of approximately 20% and 171% compared with those in 2020 and 2023, respectively. Although chlorophyll-*a* concentrations generally increased over the study period, relatively low concentrations were observed at depths where jellyfish were mainly distributed. Jellyfish tended to inhabit mid-water layers characterized by thermoclines or haloclines formed by temperature and salinity gradients rather than surface layers with high chlorophyll-*a* concentrations. No clear correlations were observed between chlorophyll-*a* concentrations and jellyfish habitat depths, suggesting that factors other than chlorophyll-*a* concentrations exert a relatively stronger impact on jellyfish distribution depths.

**Figure 10:**
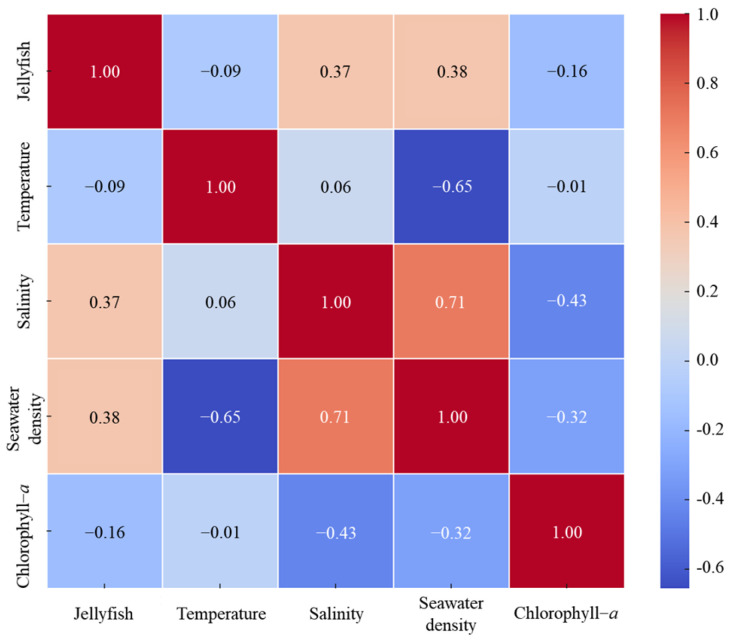
Correlation and significance analysis of *N. nomurai* count and marine environmental factors.

Correlation analyses were performed between jellyfish abundance and various marine environmental variables, including water temperatures, salinity, seawater density, and chlorophyll-*a* concentrations. The results are presented as a correlation matrix (Figure 10), along with the corresponding *p*-values, to assess the statistical significance. The analysis revealed positive correlations between jellyfish occurrence frequency and salinity (r = 0.37) and seawater density (r = 0.38), indicating that jellyfish abundance increases with increasing salinity and density. These relationships were highly statistically significant, with *p*-values of 4.85 × 10^−7^ and 2.31 × 10^−7^ for salinity and seawater density, respectively (<0.01).

### 3.3. Annual Size Distribution of N. nomurai

In May 2020, the size of jellyfish ranged from a minimum of 3.0 cm to a maximum of 60.0 cm, with an average size of 16.5 cm. In July 2020, the jellyfish sizes ranged from 25 cm to 100 cm, with an average of 56.6 cm.

In May 2021, the sizes ranged from 7.0 cm to 59.0 cm, with an average of 20.5 cm; however, in July 2021, the sizes ranged from 15 cm to 92 cm, with an average of 43.8 cm.

The sizes of jellyfish collected in May 2022 ranged from 15 cm to 100 cm, with an average of 20.5 cm. In July 2022, the specimen sizes ranged from 33 cm to 150 cm, with an average of 81.0 cm. Overall, jellyfish with an average size of approximately 20 cm and 50 cm were observed in May and July, respectively. Notably, the average size of the jellyfish observed in July 2022 was approximately 80 cm, which was more than 1.5 times larger than those collected in the previous July surveys.

In 2023, only two jellyfish were collected during the trawl surveys in May and July. However, owing to the poor condition of the specimens, size measurements were not performed.

**Figure 11:**
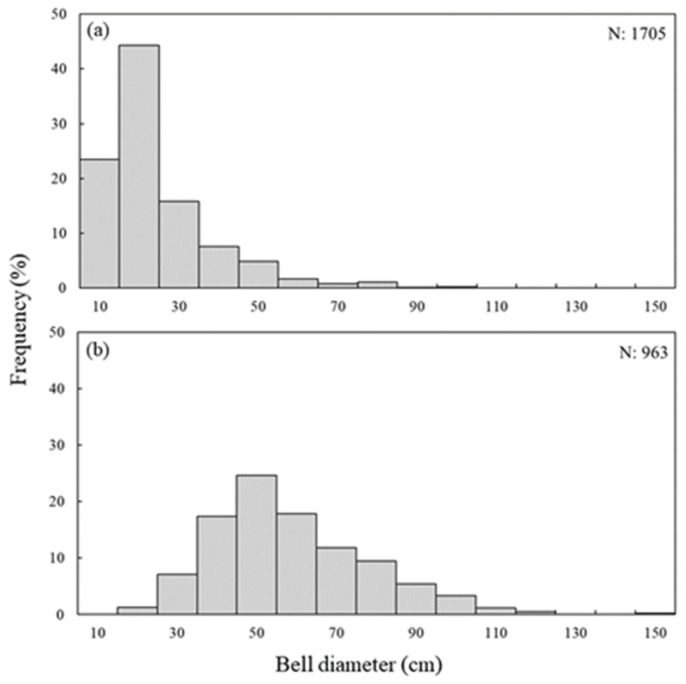
Monthly size frequency distribution of *N. nomurai* collected by trawl survey during a 5 year; (**a**) May; (**b**) July.

In May 2024, 395 jellyfish individuals were collected. Their sizes ranged from 3.5 cm to 29.0 cm, with an average size of 12.9 cm, representing the smallest average size observed over the 5-year study period. In July 2024, the jellyfish sizes ranged from 21 cm to 143 cm, with an average of 80 cm. A comparison between the smallest jellyfish individuals collected in May and the largest jellyfish individuals collected in July revealed a size difference that exceeded 40-fold (Table 4; Figure 11).

### 3.4. Analysis of Variations in the Vertical Distribution of N. nomurai and Chlorophyll-a Concentrations from 2020 to 2024

In this study, we analyzed the variations in the distribution of *N. nomurai* and chlorophyll-*a* concentrations from 2020 to 2024. The results revealed that, in areas with high chlorophyll-*a* concentrations, the primary habitat depths of jellyfish did not consistently coincide with the vertical distribution of chlorophyll-*a*. For example, in 2024, the highest chlorophyll-*a* concentrations (5.98 µg·L^−1^) were observed at a depth of 20 m. However, jellyfish did not predominantly inhabit this depth layer, which indicates that chlorophyll-*a* concentrations may not be a direct determinant of the primary vertical distribution of jellyfish.

**Figure 12:**
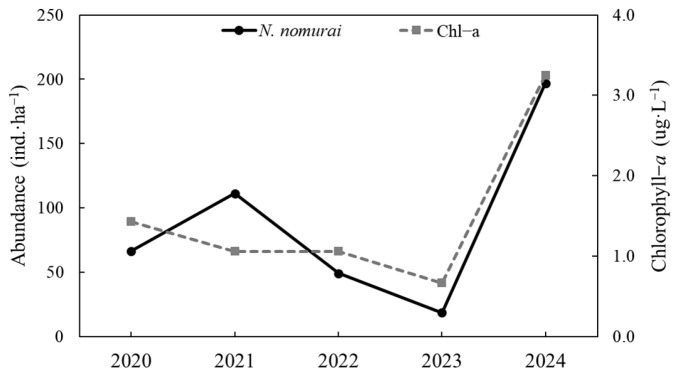
Annual variations in the abundance of *N. nomurai* and chlorophyll-*a* from 2020 to 2024. (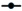: Density of *N. nomurai*; 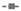: Concentration of Chlorophyll-*a*).

Nevertheless, as *N. nomurai* primarily feed on zooplankton, the distribution of chlorophyll-*a* concentrations can serve as an indirect environmental factor explaining habitat suitability for jellyfish. A comparative analysis of jellyfish abundance with chlorophyll-*a* concentrations from 2020 to 2024 revealed that both chlorophyll-*a* concentrations and jellyfish abundance increased in 2021 and 2024, whereas both variables decreased in 2022 and 2023. In 2024, chlorophyll-*a* concentrations reached their highest value (3.25 µg·L^−1^), and jellyfish abundance was also the highest at 197 ind·ha^−1^ (Figure 12).

## 4. Discussion

The vertical distribution of gelatinous organisms is closely related to the density stratification formed by differences in temperature and salinity, and jellyfish have been reported to concentrate within specific depth ranges where such stratification is pronounced [13]. These distributional characteristics can be attributed to jellyfish inhabiting physically stable water layers. Based on the density ratios reported for *N. nomurai* individuals with bell diameters ranging from 37 cm to 53 cm [10], and assuming a typical seawater density of approximately 1025 kg·m^−3^, the internal body density of jellyfish was estimated to be approximately 1029.1 kg·m^−3^. The tendency of jellyfish to be distributed mainly within layers at specific depths was inferred from the similarity between their internal body density and the density of the surrounding seawater. Marine organisms, such as jellyfish, are strongly impacted by buoyancy resulting from density differences; therefore, they tend to inhabit water layers with densities similar to their own body density, which enables them to maintain stable positioning. This behavior can be interpreted as an adaptation to enhance energy efficiency. It has been reported that *Cyanea nozakii* swim within water layers with densities similar to their internal body density, thereby minimizing their energy consumption [14]. Previous studies [15,16] have reported that *Aurelia aurita* s. l. tends to remain within water layers that exhibit seawater densities similar to their internal body density and that such distributional characteristics result from the restrictions on vertical movement imposed by density stratification. These findings provide important evidence explaining the depth-specific concentrations of jellyfish observed in the present study. Based on this hypothesis, the internal body density of jellyfish falls within a range comparable to the seawater densities observed in the study area. The internal body density of *N. nomurai* was estimated to be approximately 1029.0 kg·m^−3^, whereas the seawater densities in regions where jellyfish were predominantly distributed ranged from 1024.1 kg·m^−3^ to 1025.6 kg·m^−3^. This similarity in density enables jellyfish to remain stably suspended at specific depths, as buoyant forces that would otherwise cause upward or downward movement are minimized when the density differences are small, thereby reducing energy expenditure in the water column. Jellyfish primarily swim using jet propulsion, which generates thrust by expelling water to enable movement [17,18,19,20]. The results of this study indicate that density differences are formed at specific depths, owing to the effects of temperature and salinity. Particularly, in the mid-to-lower water column at depths of 40–60 m, density exhibited relatively uniform and stable characteristics. These stable density layers provide ideal conditions for jellyfish to inhabit without being displaced vertically by external factors. Consequently, jellyfish residing within such water layers experience reduced positional variability caused by environmental impacts, and these conditions create favorable environments for conserving energy and enhancing survival. These results suggest that jellyfish experience restrictions in vertical movement imposed by density stratification and may consequently form stable distributions by remaining within water layers with seawater densities similar to their internal body density [21]. This depth layer corresponds to a high-salinity water mass wherein salinity and density are consistently maintained and are relatively less impacted by freshwater discharge from the Yangtze River, resulting in a stable habitat. Therefore, the predominant distributions of jellyfish at specific depths are considered to be the result of multiple interacting ecological factors, including energy efficiency, habitat stability, and food availability. In addition, the predominance of jellyfish in surface waters observed during the visual surveys is likely attributable to the concentration of phytoplankton near the surface, supporting the higher abundance of zooplankton serving as prey, indicating that jellyfish ascend toward the surface for feeding. However, jellyfish that ascend to surface waters for feeding are presumed to descend to deeper layers with seawater densities similar to their internal body density after feeding to utilize the acquired energy relatively more efficiently, suggesting a survival strategy aimed at maximizing energy efficiency. While physical oceanographic factors primarily govern the vertical distribution of jellyfish, biological factors associated with food availability should also be considered, as they may influence habitat selection through trophic interactions. Chlorophyll-*a* does not represent a direct food source for *N. nomurai*, as this species primarily feeds on zooplankton rather than phytoplankton. In this study, chlorophyll-*a* was therefore used as an environmental proxy reflecting overall ecosystem productivity and trophic conditions, rather than as a variable directly determining jellyfish distribution. Chlorophyll-*a* serves as an indicator of phytoplankton biomass, which represents the primary producers in marine ecosystems, and also plays a crucial role in determining zooplankton abundance [22]. Higher chlorophyll-*a* concentrations generally indicate greater phytoplankton biomass, which can help improve feeding conditions for zooplankton and positively impact the overall structure of the marine food web [23]. Therefore, the results of this study indicate that, rather than exhibiting a direct correlation between jellyfish abundance and chlorophyll-*a* concentrations, areas with high chlorophyll-*a* levels may indirectly contribute to favorable feeding conditions by supporting higher trophic levels, particularly zooplankton, which constitute the primary prey of jellyfish. Furthermore, changes in the habitat depths of jellyfish may represent adaptive behaviors related to energy efficiency mediated by density differences. These results indicate that the vertical distribution of *N. nomurai* is governed by a combination of physical and biological influences [24]. Such characteristics constitute critical factors in explaining the ecological position and role of jellyfish within complex marine food webs. In conclusion, variations in chlorophyll-*a* concentrations can be regarded as among the key indirect factors determining jellyfish habitat conditions, as the vertical distribution and habitat strategies of jellyfish may be regulated by food availability. These findings provide valuable insights into the ecological adaptations of jellyfish and can be used to predict changes in jellyfish abundance in response to marine environmental variability.

## 5. Conclusions

This study examined the relationship between the interannual distribution patterns of *N. nomurai* in the East China Sea and marine environmental factors. The results showed that *N. nomurai* is primarily distributed in the mid-to-lower layers (40–60 m) and was highly concentrated in areas characterized by stable, high-density water masses. These findings demonstrate that physical oceanographic factors, such as seawater density and salinity, play a significant role in determining the vertical distribution of jellyfish. The preference for specific density layers suggests that jellyfish distribution is closely related to energy-efficient habitat selection under stratified environmental conditions. Furthermore, this study provides new insights into the role of biological factors by demonstrating that while chlorophyll-*a* does not directly determine jellyfish distribution, it can indirectly influence habitat suitability through trophic pathways. Higher concentrations of chlorophyll-*a* increase the availability of zooplankton, thereby creating favorable feeding conditions for jellyfish. This integrated understanding advances current knowledge of jellyfish ecology by linking physical and trophic processes in habitat selection.

Furthermore, these findings have practical implications for predicting jellyfish mass outbreaks and improving ecosystem-based management strategies, particularly in regions affected by environmental variability and climate change.

## Figures and Tables

**Table 1:** Annual and monthly dB-difference ranges of *N. nomurai* for extracting acoustic signals from acoustic data collected by a scientific echosounder.

Range of ∆MVBS_120–38 kHz_		
2020	May	[−5.84~14.05]
	July	[2.15~17.44]
2021	May	[−0.22~13.94]
	July	[4.84~16.89]
2022	May	[4.84~17.44]
	July	[10.08~20.13]
2023	May	[0.67~11.01]
	July	[10.08~20.13]
2024	May	[−5.84~9.45]
	July	[8.08~19.82]

**Table 2:** Parameter setting of single target detection algorithm applied to extract and analyze acoustic signals of *N. nomurai*.

Parameter	Values
TS threshold (dB)	−65
Pulse length determination level (dB)	6.0
Minimum normalized pulse length (ms)	1.5
Maximum normalized pulse length (ms)	7.5
Maximum beam compensation (dB)	2.0
Minor axis angles (degrees)	0.4
Major axis angles (degrees)	0.4

**Table 3:** Range and average values of marine environments at the depth of *N. nomurai* distribution from 2020 to 2024; Temperature (°C) Salinity (psu), Seawater Density (kg·m^−3^), Chlorophyll-*a* (μg·L^−1^).

Year	Temperature (°C)		Salinity (psu)		Seawater Density (kg·m^−3^)		Chlorophyll-*a* (μg·L^−1^)	
	Range	Avg. *	Range	Avg. *	Range	Avg. *	Range	Avg. *
2020	12.9~15.7	14.2	31.9~34.2	32.8	1023.7~1025.6	1024.6	0.3~2.0	0.8
2021	12.3~16.0	14.1	32.2~34.5	33.1	1023.9~1026.0	1024.9	0.3~1.4	0.8
2022	12.5~19.1	14.8	32.3~33.2	32.7	1023.7~1025.0	1024.3	0.2~1.5	0.6
2023	11.6~19.7	13.9	32.4~34.1	32.1	1024.2~1025.4	1024.9	0.1~0.8	0.4
2024	11.7~15.5	13.7	31.9~34.1	33.0	1024.1~1025.6	1025.0	0.1~2.0	0.5
Total	11.6~19.7	14.2	31.9~34.5	32.9	1023.7~1026.0	1024.7	0.1~2.0	0.6

* Avg.: Average.

**Table 4:** Monthly bell diameter (cm) of *N. nomurai* collected by trawl surveys from 2020 to 2024.

Period		2020		2021		2022		2023		2024	
	B.D. * (cm)	May	July	May	July	May	July	May	July	May	July
Minimum		3	25	7	15	15	33	nd *	nd *	3.5	21
Maximum		60	100	59	92	100	150	nd *	nd *	29	143
Average		16.5	56.6	20.5	43.8	20.5	81.0	nd *	nd *	12.9	79.9

* B.D.: Bell Diameter, * nd: No data.

## Data Availability

The original contributions presented in this study are included in the article. Further inquiries can be directed to the corresponding author.

## Abbreviation

The following abbreviations are used in this manuscript:

*N. nomurai*	*Nemopilema nomurai*
